# “Non-palliative care” – a qualitative study of older cancer patients’ and their family members’ experiences with the health care system

**DOI:** 10.1186/s12913-018-3548-1

**Published:** 2018-09-29

**Authors:** Marianne Fjose, Grethe Eilertsen, Marit Kirkevold, Ellen Karine Grov

**Affiliations:** 1 Faculty of Health and Social Sciences, Department of Health and Caring Sciences, Western Norway University of Applied Sciences, Postboks 7030, 5020 Bergen, Norway; 2 Faculty of Health and Social Sciences, Department of Nursing and Health Sciences, University College of Southeast Norway, Drammen, Norway; 3 Faculty of Medicine, Department of Nursing Science, University of Oslo, Oslo, Norway; 4 Faculty of Health Sciences, Institute of Nursing and Health Promotion, Oslo Metropolitan University, Oslo, Norway

**Keywords:** Palliative care, Older, Cancer, Family caregivers, Family research, Health services

## Abstract

**Background:**

Among all cancer patients in the palliative phase, ¾ have reached the age of 65. An aging population will increase the number of people afflicted with cancer, and create challenges for patients, family members and health services. Nevertheless, limited research has focused explicitly on the experiences and needs of older cancer patients in the palliative phase and their families. Therefore, the aim of this study is to explore what older home dwelling cancer patients in the palliative phase and their close family members, as individuals and as a family, experience as important and difficult when facing the health services.

**Methods:**

We used a qualitative descriptive design. Data was collected through family group interviews with 26 families. Each interview consisted of an older home dwelling cancer patient and one to four family members with different relationships to the patient (e.g. spouse, adult children and/or children-in-law). Data was analysed by qualitative content analysis.

**Results:**

The main theme is “Non-palliative care” – health care services in the palliative phase not tailored to family needs. Three themes are revealed: 1) exhausting cancer follow-up, 2) a cry for family involvement, and 3) fragmented care.

**Conclusion:**

The health services seem poorly organised for meeting the demands of palliative care for older home dwelling cancer patients in the palliative phase and their family members. Close family members would like to contribute but health services lack systems for involving them in the follow-up of the patient.

## Background

Among all cancer patients in the palliative phase, ¾ have reached the age of 65 [1]. The ageing population increases the number of people afflicted with cancer [2, 3] and creates challenges for patients, families and the health care system. Due to frailty and comorbidity, older patients may need extended help [4]. For patients to remain at home, particularly in the palliative phase of their illness, family members who are willing and able to provide care are important [5]. Particularly, available adult children and/or children-in-law may be important because the patient’s spouse might also be frail or deceased [6]. Thereby, the entire family may be affected [7, 8]. Despite these facts, there is limited research on the specific experiences and needs of cancer patients older than 65 years and their family members during the patients’ palliative phase [8, 9].

The goal of palliative care is to promote quality of life for patients and their families [10], which is in line with families’ wishes for a positive final time together [8]. To achieve this goal, relief of the patient’s physical, psychosocial, and spiritual symptoms and support for families is central. As in other Western countries, such as the USA and the UK, palliative care in Norway is highly developed and integrated into mainstream service provision [3, 11]. This means that palliative care should be offered wherever there may be patients with palliative care needs. Patients with cancer in the palliative phase may need regular follow-up to control symptoms and keep their cancer at bay [12]. Usually, this follow-up is monitored from hospital outpatient clinics [4, 13], such as in Norway [14]. Consequently, patients spend most of their time at home, where home care nurses (HCNs) and general practitioners (GPs) are responsible for palliative care [3, 15].

Studies have documented that older cancer patients in the palliative phase are not prioritised for palliative care [2, 9]. In addition, it is documented that older patients often have to end chemotherapy due to side effects [16, 17]. Five studies [18–22] have focused specifically on older patients’ and/or their family members’ experiences with the health care system. In other studies, wider ranges of patient’s have been studied, but the findings do not indicate any age effects related to health care system experiences [23, 24].

It seems well documented that many cancer patients in the palliative phase and their family members miss information about the illness situation, e.g., [25–27]. Information is important for family members to cope and feel safe with caregiving [25, 28, 29]. In addition, patients’ and family members’ dissatisfaction with coordination and continuity of care from hospitals and HCNs is well documented [19, 24, 30]. Often, family members report that they were left to coordinate care themselves [31, 32]. Additionally, studies document family members’ need for support from a skilled person who knows the health care system and the family and who can coordinate and control the situation [13, 24, 33]. Some specialist palliative care interventions offer families continuity and coordination [34, 35]. Preferences regarding the professionals’ personality or quality of the relationship (e.g., respect, kindness, trust, patience, and empathy) are also highlighted [23, 24, 36].

The need for family members to be “part” of the health care team is reported by both patients and family members [22, 28, 37, 38]. In addition, studies report that patients’ preferences regarding their own and/or their family members’ involvement in decision-making seem to vary and to change with time [39–41].

When living at home, access to health care services when needed is important [23, 30, 42]. It is also important that the services offered address the needs of patients and family members [23, 29, 43]. The older patients in Devik et al.’s (2015) study reported that HCN played an important role in provisions of palliative care. HCN may also ease caregiver burden [23, 31, 43]. However, help from HCNs can conflict with the need for independence [18, 23], dignity and the protection of the more private aspects of life [43]. Several studies reported that patients and family members perceived their GP to lack knowledge about cancer treatment and therefore preferred to turn to their specialist at the hospital when problems arose [24, 44, 45]. For patients living in rural areas, travel to hospitals for follow-up are reported to be long and fatiguing [21, 46, 47].

The above literature review reveals some prior knowledge about individual cancer patients’ and family members’ experiences facing the health care system during the palliative phase. The study samples comprise older patients. However, we have not found studies that have documented findings about several family members’ and the family unit’s experiences and needs regarding the health care system, as all studies included have collected data through individual or focus-group interviews with patients or family members. We need more in-depth knowledge on how families experience situations in the patients’ palliative phase in terms of organisation and collaboration with the health care team. Based on the descriptions and suggestions gathered from the older cancer patient and his/her family, strategies to support families in ensuring a positive final time together should be identified. The aim of this study is thus to explore what older home-dwelling cancer patients in the palliative phase and their close family members, as individuals and as a family, experience as important and difficult when interacting with health services.

## Methods

The study has a qualitative design. Data were collected through family group interviews and analysed using qualitative inductive content analysis.

### Sample and recruitment

A purposive sample of families was recruited. Nurses in community health services, hospital wards and outpatient clinics were asked to distribute information on the study, which included information about the researchers’ goals, and to recruit patients and their family members according to the recruitment criteria. Families willing to participate either returned written consent forms or gave the nurse permission to pass on their telephone number to the interviewer. The patient or the person the family had listed as a contact person was then contacted and, if desired, given additional information on the study. The inclusion criteria were cancer patients in the palliative phase ≥65 years and their close family members ≥18 years. The included patients were required to be living at home, capable of providing informed consent and capable of participating in group interviews. Small groups of no more than 5 participants were chosen due to the families’ vulnerable situations [48], because families are complex systems [49] and because it was important that the interviewer be able to lead the interviews while simultaneously observing the interactions and processes among the participants [50]. As we wanted to recruit family members with a variety of relations to the patient, an open definition of the concept of family was adopted: “The family is who they say they are” [7]. To ensure a variety of relations in the recruitment, the interviewer discussed with the family contact person which family members were to participate, e.g., to secure sons and children-in-law in the sample. However, the final decision was left to the family.

### Data collection

In family group interviews, data are collected on family members’ individual experiences and opinions as well as the family’s shared experiences and opinions [7, 51, 52]. There is very little theory available on interviewing families as a group; thus, in line with other family researchers [51], we looked to focus-group methodology for guidance. Some of the principles and understandings that focus-group research is based on are transferable to interviews of family units. However, family members are related to one another through the past, the present and the future and know each other intimately, which is generally not the case for focus-group participants [53]. As we had small groups, and the interviewer had prior experience with qualitative interview research, all interviews except one were carried out without a co-moderator [53]. The last author participated as a co-moderator in one interview to guide the interviewer.

The interviews were carried out in the patients’ homes and generally started with the drawing of a genogram, which is a diagram of the family constellation [7]. If the participants were eager to start talking, the genogram was postponed until a natural break appeared or at the end of the interview. The purpose of the genogram was to collect data on the family’s structure and to have the participants reflect on family relations (7). The interviews were based on a thematic interview guide that was developed by the authors and pilot-tested with one family. The opening question was: “How are you doing now, each of you?” Additional questions such as “How have you experienced your encounters with the health services?” and “How can the health services support your family now?” were asked. Circular questions focusing on cognitive, behavioural and emotional areas, such as “What do the rest of you think regarding what … now says?” were used to stimulate reflections and collect data on various family members’ experiences and needs [7]. The interviews ran similar to a conversation where participants and interviewer collaborated on constructing meaning from questions and answers [54], and follow-up questions were asked by interviewer and participants to explore themes and clarify the content of what had been said.

There was typically no spokesperson in the families, possibly because the interviewer at the start of each interview told the families that both individual and common opinions and experiences are generally present in families, and the objective was to hear all of these opinions. However, some patients would at times need some help from family members to explain what they were trying to say. Due to frailty, a few patients also rested at times while the family members talked. The interviews lasted from 90 to 120 min and were audio-recorded. The first author transcribed the first 15 interviews, and due to time constraints, the last 11 interviews were transcribed by a hired person who signed a contract, consenting that the data would be treated with confidentiality and not shared or otherwise communicated to others.

The interviewer attempted to be sensitive to and respectful of the families’ situations, emotions and reactions during the interviews. There was laughter as well as weeping, and the interviewer respected the participants if some subject was clearly avoided.

### Analysis

The data were analysed using inductive content analysis, which means that codes were created openly from the text, and categories were freely generated, not from predetermined coding sheets. Findings were related to both manifest and latent content in the material [55, 56]. The transcribed text was first read repeatedly to gain familiarity with the text and an idea of the whole picture. The text was subsequently divided into units of meaning, all with content relevant to the purpose of the study. By posing the question “What does this text say about the patients and/or the family members’ encounter with the health services?” the units of meaning were abstracted and openly coded. The codes were then evaluated in terms of differences and likenesses and categorised to create groups matching these perspectives. The groups were given names, i.e., to create categories. These categories mainly addressed the manifest content [55, 56]. To identify the latent content, the categories were further interpreted. Statements in each category were read critically and compared with other categories, codes, units of meaning and the transcribed text as a whole, and they were subjected to the following questions: “Are the experiences and opinions of the patients and the family members similar or not?” and “What is happening in the family relationships here?” Through this process, the categories were condensed into one main theme and three subthemes that described the underlying, abstract meaning of the text as a whole (see Table 1).

**Table 1:** Example of the analysis process

Theme	Exhausting cancer follow-up		A cry family involvement		Fragmented care
Categories	Patient follow-up	Exhausting traveling	Family follow-up	Information	Primary health care
Codes	Disease history	Patient travels	Knowing the system	No answers	Home care
	Elderly in hospital	Obligation to work	Advocate	Have to nag	Cancer nurses
	To be taken seriously	Latency, waiting time	Work responsibility	Allowed to ask	General practitioner
	Staff competence		Family as health care personell	Honest information	Contact
	Trail treatment plan		Family responsibility	Understandable information	Respect
	Stigmatisation		Attitudes towards health care	Different information	To be seen
			System mess	Good information	Taken seriously
			Bureaucracy	Disease information	
			Observation		

### Trustworthiness

To convey the trustworthiness of this study, the entire research process, including recruitment, sample, data collection and data analysis, is described in detail. Conducting 26 family group interviews yielded rich and comprehensive data. The data seemed well saturated, and the final interviews did not provide new insights, i.e., no new topics evolved through the final interviews. All the authors were female and had a preunderstanding as nurses and researchers of geriatric and cancer care and as family members. Before starting the data collection, the interviewer (the first author) reflected on her own preunderstanding of families and family relations in a palliative phase. Reflexive notes were written immediately following each interview, while listening to the interviews, during transcription and during the analysis process, with a focus on both the interviewer’s role in the interviews and on the content of the interview conversations. The last 11 interviews, which were transcribed by the hired person, were carefully listened to in their entirety to ensure correct transcription. Elo et al. (2014) suggest that one researcher be responsible for the analysis. In this study, the first author was responsible for developing the process of analysis from codes to categories to themes. Then, all authors met, and the tentative codes, categories and themes were discussed and revised several times until a consensus was reached. Findings are presented with support from representative quotes to give the reader the chance to evaluate alternative interpretations. The family group interviews were conducted in Norwegian. To ensure the correct translation of quotes when transferring from spoken words during the interview to the transcribed text and to the English translation for this manuscript, the quotes were translated back to Norwegian for review by all four authors separately.

## Results

Thirty-three families agreed to participate in this study. However, due to patients’ deteriorated health conditions, seven families withdrew before the interview. Because the nurses who helped with recruitment approached the patients, we have no list of the families that refused to participate. The final sample consisted of 26 ethnic-Norwegian families. A total of 86 people participated in the study. See Table 2 for demographic details. Nine patients lived alone, 2 with one of their children, and the others lived with their spouse. One spouse did not participate due to poor health. Every family group interview consisted of the patient and 1 to 4 family members, with an average of 3 participants per interview. For details on the patients’ cancer diagnoses and patients’ and spouses’ other diagnoses, see Table 3.

**Table 2:** Sociodemographic characteristics of participants

Characteristics	Mean	Range
Family Members age (*N* = 86)		
Patients	79	(65–92)
Spouses	73	(55–84)
Sons	49	(33-56)
Daughters	46	(27–62)
Son-in-law	63	
Daughters-in-law	45	(34-53)
Grandchild	21	
Sister	65	
Characteristics	n	%
Relation of Family Member to Patient (*N* = 60)		
Wife	10	16
Husband	4	7
Son	11	18
Daughter	26	43
Son-in-law	1	2
Daughter-in-law	6	10
Grandchildren	1	2
Sister	1	2
Children’s Marital Status (*N* = 37)		
Paired	24	65
Non-paired	13	35
Family Members’ Educational Level (*N* = 60)		
High school and above	31	52
Below high school	29	48
Family Members’ Employment Status (*N* = 60)		
Employed full-time or part-time	45	75
Retired or on sick leave or not employed	15	25

**Table 3:** An overview of patients’ and spouses’ self-reported diagnoses

Patients				Spouses
Cancer type		Other diseases		Other diseases
Lung	7	Cardiovascular diseases	10	4
Prostata	6	Polymyalgia	1	
Breast	1	Hearing impairment	4	1
Colon	6	Musculoskeletal diseases	9	6
Amyliose	1	Mental disease	1	
Gynaecological	1	Cerebral diseases	4	2
Mole	2	Renal failure	1	1
Bone marrow	1	COPD, asthma	3	
CML	1	Diabetes	2	2
Myelomatosis	1	Vision impairment	1	
Unknown	1	Crohn’s syndrome	1	

Many patients in this study had reduced physical capacity and some reduced mental capacity. As a result, the hospital, the HCN and the GP were often involved in the follow-up of the patients. What was important for the families in their encounters with health services was also often the most difficult, and negative experiences were more apparent in the conversations than positive experiences. The main theme in the study was “non-palliative care” – health care services in the palliative phase not tailored to family needs. The findings were divided into 3 themes: 1) exhausting cancer follow-up, 2) a cry for family involvement, and 3) fragmented care.

The findings illuminated the patients’ and family members’ individual opinions and experiences, the families’ common experiences and the interactions within the families. The families are designated A to Z.

### Exhausting cancer follow-up

Even if some patients were able to handle the cancer follow-up at the hospital well, follow-up proved exhausting for the frailest patients. Several families said the patient had become so weak in connection with tumour-directed palliative treatment that he or she had to spend 1–3 months in bed in a hospital or nursing home. Travelling to the hospital was also quite an ordeal for the frailest patients due to long distances, reduced mobility and painful symptoms. Some patients and family members thus cancelled, or did not book, follow-up appointments so that the patient would not have to travel. The following quotes from two families illustrate the level of exhaustion the cancer follow-up could cause:

Son: *«However, it was when he was given chemotherapy that he started going downhill.»*

Wife: *«Then, he was really sick; he couldn’t even stand on his own two feet. He was in the hospital and the nursing home for almost 3 months»* (family K)*.*

Daughter: *«You [the patient] avoid contacting the health services; it is just so cumbersome and difficult. I haven’t pushed for hospital involvement either. I just think, no, it’s too difficult, and some appointments have been cancelled»* (family B).

Some patients and family members said it was better to have rewarding time at home than to suffer away from home. Other family members, however, found it disturbing not to know how the cancer was developing. The following quotes illustrate this:

Wife: *«He said “No” to more therapy. The hospital called him in three times, however, and he refused, he did not want to go. He may not have lived had they convinced him to receive more treatment»* (family K)*.*

Daughter: *«It would have been nice if Mom had some follow-up to see if her cancer was spreading. We don’t know anything.»*

Husband: *«No, but I guess they figured that she would be better off doing nothing. Because then you have to travel and … that’s exhausting»* (family T).

A few families had good experiences with follow-up from the GP. However, most families struggled, as most GPs in Norway do not make house calls and getting to the GP’s office could be an ordeal for frailer patients, as one daughter states in the following:

*«And it is not all that easy to get to the doctor’s either. I really wish there was some old-fashioned family doctor available, making house calls again»* (family B).

Several families expressed low confidence in the GP’s competence and preferred to contact the hospital when problems arose. Low confidence seemed to be linked to experiences with the GP initially not taking the patient’s complaints seriously, resulting in a delayed diagnosis. Some families thus blamed the GP for the patient’s poor prognosis. The following quotes illustrate this:

Patient: *«The doctor just does not really know anything about this; you should keep far away from him. You need to go to where the expertise is»* (family M).

Husband: *«She’s very bitter, or angry, at her doctor.»*

Patient: *«He should have seen this a long time ago»* (family Q)*.*

When patients were unable to take a private car to the hospital, publicly organised “collective taxis” were used. These journeys were quite exhausting to many of the patients and were deemed “horrible” (patient U) and “torture” (patient M). These collective taxis must be filled, and up to 4 patients were collected at various addresses. As the following quote illustrates, the whole day may be spent travelling:

Son: *«They pick them up, driving miles and miles here, then get on the ferry and into town. Then, they may have to wait for an hour or so until all are done, and then they have to do the same rounds when they come home. It takes the whole day!»* (family L).

The roads in western Norway are often narrow, winding and bumpy, and during winter, they are also icy and slippery. This, in combination with full taxis, where patients who are not well and who do not know each other sit close to one another, added to the discomfort of the journeys. Such journeys may end in re-admittances, and the families wanted the patient to get a taxi straight home. The following quotes illustrate this:

Sister: *«In the summers, it is more or less ok, but these long trips in the winter are far worse, with all sorts of weather and wind»* (family M).

Wife: *«We travelled in a hot and full taxi; we sat so close. When we got home, the taxi driver and me pulled him into the house. Then it was just to call 113, and we were rushed back to the hospital to the doctor in an ambulance with sirens»* (family K).

Due to the hardship of travelling by the collective taxis, many families went to great lengths to provide transport for the patient. Six patients had spouses with sufficient health and a license to drive a car, and some children and/or children-in-law were able to take time off from work to drive the patient. As the quote below illustrates, being unable to drive the patient could be an emotional burden:

Daughter: *«And when he has to go to the doctor’s, how are we going to get that done? We are at work, of course. Therefore, we feel guilty»* (family F).

### A cry for family involvement

Many patients did not wish to, or were unable to, maintain contact with the health services and handle the information regarding their own health situation. As a result, they had more or less completely delegated that responsibility to their family members. The following two quotes and a family conversation sequence illustrate this:

Patient: *«My daughter has “taken over” me»* (family A)*.*

Daughter: *«You don’t remember well when you receive messages or things similar to that»* (family B).

Patient: *«When I’m at the doctor’s, and he tries to explain something, I hardly listen to what he says.»*

Son: *«To put it like this, he gets what they say, but he never asks.»*

Wife: *«He never asks about anything.»*

Patient: *«No, I do think they should say it as it is. But that isn’t all they say.»*

Son: *«That’s true, and there is probably a lot you want to ask about anyway?»*

Patient: *«Maybe I am just afraid of the answer»* (family L).

The family members thus had the responsibility at home to follow up any tumour-directed palliative treatment, symptom alleviation and care and to contact the health services when problems arose, coordinate appointments, and transport the patient to such appointments. Such responsibility was accompanied by the need to be involved in the health services’ follow-up of the patient. It was particularly important to be involved in the information exchange around the patient’s health situation. As the quotes below illustrate, information yielded security, control and predictability:

Daughter: *«I feel that if I’m given information, I’ll be able to handle it all a bit better»* (family E)*.*

Daughter: *«It has to do with what to expect and what to consider, and what is important now and what is not»* (family B).

The family members considered the information exchange a reciprocal process. The family members had important information on the patient’s symptoms and in-home resources, and the health services need this information to provide the patient with the best possible follow-up. The following conversation among a family illustrates this:

Daughter: *«Mom may be doing ever so poorly, and then 2 days later when she is at the doctor’s she says she’s fine.»*

Daughter-in-law: *«And then we have to ask her, …. but how about 2 days ago?»*

Daughter: *«Then, we have to remind her»* (family N).

Family members sought information first and foremost from the hospital, but it was difficult to participate in the hospital’s information exchange. Hospitals seem to primarily talk to the patients, without mapping their wishes and capacity for administering the information. As a result, as the quote below illustrates, much information is lost, which causes anxiety and unease:

Daughter: *«However, I guess they informed Mother [the patient].»*

Husband: «*Mmmm, well… I don’t know about that.»*

Patient: *«No, neither do I.»*

Daughter: *«I know. That has been part of the problem too, I guess. They have given mother information, and she hasn’t quite got it all»* (family I).

The family members were unfamiliar with the health care system and felt left out. They did not know what was expected of them, what to expect, or whom to contact. They learned to navigate the system only by being in the system and by trying and failing. They had learned that they had to take the initiative and often keep on asking, repeatedly, to get information. The following part of a family conversation and quotes illustrate this:

Eldest daughter: *«The admission system is not good enough.»*

Youngest daughter: *«You are just not met with openness, that it is similar to this or that. They just take it for granted»* (family N).

Daughter: *«I guess we weren’t good enough at asking questions … we did learn a LITTLE as we went along asking questions»* (family I).

The decision to delegate the responsibility to close family members was made differently among the various families. It seemed easier for the patients to delegate and for family members to take over when a spouse or a family member had health service training. The following conversation sequence illustrates this:

Patient: *«She [daughter-in-law] is after all a trained nurse. She is ready for action. That makes me feel safe.»*

Daughter-in-law: *«And I do ask if something comes up, it may have something to do with me having a bit more knowledge about some of these things»* (family U)*.*

As the next quote illustrates, some adult children struggled to balance the delegated responsibility with respect to the patient’s autonomy and dignity:

Daughter: *«I do feel like I’m stepping a bit too closely. This is after all confidential information»* (family B)*.*

Family members’ individual information preferences in addition to the patients’ information preferences might also influence the family members’ search for information, illustrated by the following quotes:

Daughter: *«We don’t need to know more than we need to. Therefore, we don’t get nervous»* (family A).

Son: *«We have also made a point of not asking too much, so if she asks us, we don’t have to lie to her»* (family G).

Doctor’s appointments were seen as the most secure sources of information for the family members, which made it difficult for working children and children-in-law to obtain information. As the following quotes illustrate, family members desired a programme for paid leave from work, which would enable them to follow up their seriously ill family members:

Son: «*It is not all that easy when you work. I just now received a letter that I could not take out any more paid leave»* (family H)*.*

Daughter-in-law: *«You can take out paid leave when the kids are sick, but it really should be the same when close family are sick»* (family U).

A series of suggestions for improving the involvement of family members in the exchange of information was made, such as inviting family participation, family conversations and alternative information channels:

Son-in-law: *«Some written information from the doctor would be good.»*

Daughter: *«Yes, some feedback when you have been to the hospital and that it’s given to us too.»*

Son-in-law: *«You could easily fix that confidentiality thing if he signed some letter for the family»* (family B).

Daughter: «*I guess it is fine that Mom [patient’s wife] has been there, but I’m thinking a bit further, that I, as his daughter, could also have been there. Then, you could ask about the things that you wonder about»* (family V).

### Fragmented care

Most families found the health services to be fragmented. They wished to get to know the personnel who would be following them up; thus, the help could be adapted to the patient’s and family members’ needs and be experienced as predictable and continuous. Getting to know the personnel demands continuity among the personnel and would enable the establishment of safe and trusting relations with personnel.

Lack of continuity in the oncologist at the hospital might result in the oncologist spending the consultation time reading the patient’s journal. This left little room for patients and family members to receive answers to their questions, which the following quote illustrates:

Patient: *«I never had the same doctor, I’ve been in contact with a whole series of them. In addition, then they would spend the time reading my journal. I learned nothing new about my treatment. In addition, then I just had to leave. I felt I did not really have anything to do there»* (family X)*.*

To ensure continuity in contact with the oncologist, it was important to some families that the same family members accompany the patient every time:

Daughter: *«I find it so important that the doctor knows Mom [patient]. My sister, who accompanies Mom, knows her illness history from A to Z. If I or my brother were to accompany Mom, none of us have met the doctor and we would have had to start all over again»* (family N)*.*

The families did not want HCNs to always send new people to their homes. It was particularly difficult for patients to receive care from strangers, who did not know their needs, and this meant unpredictability concerning how help would be given. The following quotes illustrate this:

Wife: «*From the HCNs, they are different every single Sunday. I do not think we would have managed if they were to be around here. However, now they don’t even come in, just hand over the pills and leave»* (family L).

Patient: *«However, I think about those nurses, they can’t really bathe me either… They can hardly touch me without me getting bruises.»*

Husband: *«She [patient] has some problems with some of the nurses … it depends on who comes whether she will let them into the bathroom with her. However, now we know who those nurses are. Therefore, now even I know who might be of great help to you»* (family Q).

Nine out of 26 families were followed up by a cancer nurse. The cancer nurse was the same over time and an important resource for the families. She communicated information and helped families navigate the health care system. Some families developed a trusting relationship with the cancer nurse, who thus became an important conversation partner during difficult times. Quotes from two families illustrate this:

Wife: *«We get on well with the cancer nurse. We can ask her for advice and she explains different things. We trust her»* (family K)*.*

Patient: «*And then she [cancer nurse] takes care of things that we can’t do, such as talk to the people at the hospital»* (family E)*.*

Fragmented and unpredictable follow-up from the HCNs made the services not very attractive for many families. As a result, family members would go to great lengths to help the patient themselves. Sometimes, when the patient would want to manage without the HCNs and the family needed relief, tensions and conflicts would arise within the family, which the following quotes illustrate:

Wife: *«He chases away the HCNs. He doesn’t want them here. I am the ‘HCN’ … »* (family K).

Husband: *«I criticise you a bit for not accepting more help from the home care services for your daily personal care.»*

Patient: *«Then, we argue a bit»* (family Q).

Some families also experienced fragmented follow-up from the GP. As the following quote illustrates, the substitute doctors who did not know the patient well were more prone to sending the patient to the hospital, which led to unnecessary, exhausting journeys:

Daughter-in-law: *«They are very quick about sending the patients on, and that may be an advantage and a disadvantage both»* (family H).

## Discussion

This study is, to our knowledge, the first study that specifically explores what home-dwelling older cancer patients in the palliative phase and several close family members, as individuals and as a family, experience as important and difficult when facing the health care system. Our findings suggest that for patients and family members in this study, the health services were poorly organised. First, the hospital’s cancer follow-up was exhausting for many patients. Second, close family members did not feel sufficiently involved in the health services’ follow-up of the patients, even if they frequently were responsible at home for keeping track of the patients’ cancer follow-up. Third, the families experienced the help from the health care services as fragmented.

### «Non-palliative care» to frail older cancer patients

For the frailest patients in this study, the tumour-directed palliative treatment was exhausting. Other studies have documented that chemotherapy has a more adverse effect on older patients than on younger patients [16, 17]. Earlier studies have also documented that older patients are not prioritised for palliative programmes [2, 9]. One study showed that palliative consultation led to a reduction in the use of chemotherapy in older cancer patients [57]. It may be particularly important that older patients receive follow-up from personnel who are competent in palliative care so that the cancer follow-up is adapted to the individual patient’s needs and resources.

The journeys to the hospital for cancer follow-up were exhausting for the patients. This finding is also reported in a study of rural Norway [21] and in studies of rural areas of Australia and Canada [46, 47]. The findings from our study are interesting because they expand existing knowledge by showing that the journeys often became so exhausting that appointments were cancelled, and the cancer follow-up was discontinued. The journeys by collective taxi were particularly exhausting. These journeys do not appear to further the intentions of palliative care. More patients could possibly have coped better with the cancer follow-up if they travelled alone in a taxi directly between the hospital and their home.

This study shows that the frailest patients and their close family members experienced the GP as difficult to access, which was also documented in a Danish study [24]. Several families in the present study had low confidence in the GP’s competence regarding the cancer follow-up, a finding also reported in several earlier studies [24, 44, 45]. It is problematic that some patients who live at home without hospital follow-up experience the follow-up from the GP as insufficient. Accessibility and competence are key factors in palliative care [58]. Care that does not include accessibility and competence may therefore be interpreted as “non-palliative care”.

### Lack of routines for family involvement

Our findings reveal a great need for the involvement of family in the health services follow-up of older patients. A number of studies have documented similar findings, both in patients [38, 41] and family members [28, 37, 38] and from the perspectives of family members of older patients [22]. The need for family involvement was in this study tied to the patients’ incapacity to personally keep track of information and contact with the health care services. The family members in Williams et al.’s (2018) study reported similar findings; they felt compelled to navigate the patient’s health services because they observed that it was challenging for the older patient to interact with the health services. It has been previously documented that older cancer patients may have difficulties remembering information and that family members help the patient recall information, ask questions and report symptoms, [39, 59, 60]. However, we have not found other studies that have reported that older patients more or less completely delegate all of their health care responsibility to close family members. This study thus shows how important it is to involve close family members in the health services’ follow-up of the patient to give the patient the best possible follow-up at home and in the health services.

The findings of this study show that hospitals seem to lack routines for mapping the patient’s wishes and the capacity for administering information and for involving the family when necessary and desired. Doctor’s appointments and controls were described by the families as the best information channels, but for working children and children-in-law, these information channels were difficult to access. Similar results have been reported previously [23, 29]. To ensure the best possible follow-up of the patients, it is important that health services develop alternative routines for information exchange and family involvement. The family members in this study suggested family conversations, routinely attending follow up appointments with the patient, and being provided written summaries from consultations. Speice et al. (2000) argued that telephone and e-mail may also be used. This must, of course, occur with the patient’s consent. In this context, however, it is important to note that patients have described positive and negative aspects of family involvement in treatment decisions. For instance, patients do not like when family members obtain information without their knowledge or consent [39]. The relatively limited knowledge on older patients’ preferences regarding family involvement indicates a need for dialogue between patients, family members and health services to map the family’s expectations and the need for family involvement.

### Lack of continuity – Lack of safety of care

The families found the follow-up to be fragmented, from the hospital oncologist from the GP in the municipality and from the HCN. Getting to know personnel, which depends on continuity in the personnel group, was important for the help to be adapted and predictable and to ensure the family felt safe. These findings have been well documented in earlier studies [29, 30, 41]. Continuity of care throughout the illness trajectory and across various levels in health services is the basis of palliative care [58]. This study suggests that the organisation of the health services is not in line with the principles of palliative care. The cancer nurse service was an exception and gave continuity and security to some families. Studies that evaluate palliative services (e.g., palliative ambulating teams, palliative nurse in the home) report that palliative services give patients and family members continuity and security [34, 35]. Several studies emphasise the significance of a contact person in a palliative phase [13, 24, 33]. A cancer nurse in municipal health services may constitute such a contact person. The reasons why not all families in this study were followed up by a cancer nurse may vary. As mentioned earlier, older patients are not prioritised for palliative care services [2, 9]. Additionally, the majority of the families were recruited from small municipalities where access to services and competence may be challenging. The goal of follow-up in the home, and the aim of palliative care, thus seems difficult to achieve in rural Norway [15], a tendency also seen in Australia and Canada [20].

### Family involvement to support family interplay

The findings in this study show that encounters with health services can become a challenge to family life. This was especially true for the passing on of the responsibility for keeping in contact with the health services to close family members, for the patients’ and family members’ various information needs, and for the patients’ and family members’ various needs for help and support from the HCN. Cancer patients’ and family members’ preferences regarding content, timing, and delivery of information may vary [25, 29]. Van Eechoud, Piers, Van Camp, Grypdonck, Van den Noortgate, Deveugele, Verbeke and Verhaeghe [61] found that when family members’ wishes for involvement in older patients’ advanced care planning did not agree with those of the patient, relations might become tense. We are not aware of other studies about family processes related to families’ encounters with health services. The abovementioned challenges connected to family life may be related to the families’ lack of knowledge of and lack of involvement in the health care system. Family members who were not trained in some health care profession did not know what was expected from them, what to expect or whom to contact. Established routines at hospitals for dialogue on expectations and need for family involvement will most likely contribute to a common understanding within the family and in meetings between the family and the health services on how the individual family should become involved to secure the best possible follow-up of the patient.

### Study strengths and limitations

We wanted to recruit families with a variety of family structures, so we chose an open definition of the concept of family. Nevertheless, only families related by blood or marriage were recruited, possibly because the nurses helping with the recruitment asked only biological families or because biological families are the most common family composition among older people in rural western Norway. This could represent a study limitation, although it was difficult to avoid because other personnel performed the recruitment.

Another possible study limitation is that the transcripts were not returned to the participants for comments, nor were the findings presented to them to invite feedback on the accuracy of our interpretations. As the participants in this study were patients in the palliative phase and their closest family members, we considered that such a request would be an extra burden for them.

This study is one of few studies in the context of advanced cancer that has used family group interviews for data collection. Family group interviews in this study contributed to a new understanding of older adult cancer patients’ and their closest family members’ individual and shared experiences and needs in facing the health care system. It has been argued that individual interviews are most effective when the topic is sensitive. The participants may then express their feelings more openly. In our study, a few family members expressed, while standing at the door after the interview, that they withheld sensitive information so as not to burden the patient. This might also be a study limitation. However, data on how the patient passed on the responsibility of their health condition to family members seemed difficult to access in individual interviews. It is also uncertain whether the frailest patients in this study would have consented to participate in individual interviews.

Only a few of the families in our sample reported extensive challenges in relation to the health care system as a result of cancer. Because participating in research is voluntary, it is possible that families with more difficult interactions with the health care system refused to participate. Consequently, the findings must be interpreted with caution and may not be generalisable to all families in similar situations to the families studied here. However, these results and reflections may be relevant to families in similar situations.

Finally, this was a cross-sectional assessment, and a longitudinal design may have provided richer data. As the participants were in a palliative care phase, we assumed that participation in several interviews would be too tiring and thereby ethically challenging.

## Conclusion

This study shows that the health services’ follow-up was poorly adapted to older cancer patients in a palliative phase and their close family members’ need for help and support. The organisation of the services, as well as access to services and competence within palliative care, need improvement. As long as these aspects do not function properly, it is difficult to reach the stated aims of palliative care. Routines for family involvement in the health services’ follow-up of the patients when needed and wanted and providing a contact person with specialist competence in the municipality who can create continuity and predictability are suggestions to ensure that older palliative-phase patients’ and their close family members’ follow-up is in line with the intentions of palliative care.

Further research is needed on the experiences and needs of older cancer patients in the palliative phase and their close family members regarding the involvement of close family members in the health services follow-up of the patient, such as concerning advanced care planning and decision-making.

## Abbreviations

GP: general practitioner
HCN: home care nurse
